# Hysteresis and Related Error Mechanisms in the NIST Watt Balance Experiment

**DOI:** 10.6028/jres.106.028

**Published:** 2001-08-01

**Authors:** Joshua P. Schwarz, Ruimin Liu, David B. Newell, Richard L. Steiner, Edwin R. Williams, Douglas Smith, Ali Erdemir, John Woodford

**Affiliations:** National Institute of Standards and Technology, Gaithersburg, MD 20899-0001; Argonne National Laboratory, Argonne, IL 60439

**Keywords:** DLC coating, hysteresis, Josephson constant, kilogram artifact, Planck constant, watt balance

## Abstract

The NIST watt balance experiment is being completely rebuilt after its 1998 determination of the Planck constant. That measurement yielded a result with an approximately 1×10^−7^ relative standard uncertainty. Because the goal of the new incarnation of the experiment is a ten-fold decrease in uncertainty, it has been necessary to reexamine many sources of systematic error. Hysteresis effects account for a substantial portion of the projected uncertainty budget. They arise from mechanical, magnetic, and thermal sources. The new experiment incorporates several improvements in the apparatus to address these issues, including stiffer components for transferring the mass standard on and off the balance, better servo control of the balance, better pivot materials, and the incorporation of erasing techniques into the mass transfer servo system. We have carried out a series of tests of hysteresis sources on a separate system, and apply their results to the watt apparatus. The studies presented here suggest that our improvements can be expected to reduce hysteresis signals by at least a factor of 10—perhaps as much as a factor of 50—over the 1998 experiment.

## 1. Introduction

In the realms of metrology and fundamental constants, an important role is played by watt balance experiments based on the method suggested by Kibble [1]. A watt balance measurement conducted at the National Institute of Standards and Technology (NIST) is responsible for the most accurate measurement of the Planck constant, and has reduced the uncertainty in many physical constants. These include the Josephson constant, the charge and mass of the electron, and the Avogadro constant [2]. Currently the NIST experiment is undergoing major redesign as we attempt to reduce uncertainty by an order of magnitude—i.e., to the level of 1×10^−8^ relative standard uncertainty [3]. This paper presents information about a specific set of error sources in the NIST experiment—those resulting from hysteresis mechanisms. Although the studies presented here are specific to the NIST experiment, some of them do relate to other implementations being carried out in Great Britain and Switzerland [4,5].

### 1.1 Background Information

Since the development of the International System of Units (SI), many experiments have been developed in order to realize SI units to ever lower levels of uncertainty. Improving accuracy is an important aim for experimentalists to pursue—only at the level that the units have been realized can one compare different experimental measurements of fundamental constants. Such comparison is itself a worthy goal because increasingly varied and rigorous tests of physical theories form the basis of the physicists’ creed.

A good example of an experiment developed to realize the SI base unit of current is the ampere balance [6]. In an ampere balance the force between two well-characterized current carrying coils is measured. One coil is suspended from a balance; this is called the force coil. When current flows in the force coil, it interacts with the magnetic flux gradient created by current flowing in the second coil, creating a force between the coils. This force can be measured by comparing it to the weight of a well known mass (assuming that the local acceleration of gravity is well known). The magnetic interaction is highly dependent on the geometry of the coils. The ratio of the force *F* produced by the coils to the current *I* flowing in them, is proportional to a factor *β* that describes the geometric dependencies of the apparatus:

F/I=β. (1)

By measuring the geometry of the coils, the SI value of the current flowing in the windings can be deduced from the Biot-Savart law and the SI definition of the ampere:

The ampere is that constant current which, if maintained in two straight parallel conductors of infinite length, of negligible circular cross-section, and placed one meter apart in vacuum, would produce between these conductors a force equal to 2×10^−7^ newton per meter of length.

Historically ampere balances were also used to measure the unit of voltage in the United States; if a known current flows through a known resistance then a known voltage drop is generated. Resistance in the SI unit ohm can be measured, for example, with a calculable capacitor experiment [7].

Ampere balances have been in use for nearly a hundred years, but suffer from serious shortcomings. The most important weakness of the ampere balance experiment is its reliance on measurements of coil geometry—determining *β* is the limiting factor of the experiment. It is very difficult to measure coil dimensions, and to maintain those dimensions, at low levels of uncertainty.

### 1.2 The Watt Balance Method

In the early 1960s the landscape of current and voltage metrology changed. The experimental development of the Josephson effect allowed voltages to be linked to unchanging fundamental constants with extremely high precision and stability [8]. Later, in the 1980s, the quantum Hall effect played a very similar role in improving resistance metrology. Unfortunately the values of the voltages produced by the Josephson effect were not known with reduced uncertainty in terms of the SI volt. Strengthening the link of the Josephson voltage standard to the SI system was a task that has been dominated by watt balance experiments.

Watt balances are, to first approximation, nothing more than ampere balances with an additional calibration step that permits the removal of dependence on geometry. This calibration step incorporates a high quality voltage reference (in practice a Josephson voltage standard), and uses the force coil as an induction coil. If the force coil of an ampere balance is moved at constant speed *v* through the magnetic flux gradient produced by the second coil, then a voltage *V* is induced across it. The ratio *V/v* is related to the geometry of the experiment in the same way as the *F/I* ratio:

V/v=β. (2)

This is the calibration measurement of *β*. Because *V/v* is much easier to measure accurately than the actual geometry, watt balance experiments have a great advantage over ampere systems. Note that although direct measurement of geometry is unnecessary, the short-term stability of the system geometry is assumed—this is an assumption touched upon in Sec. 3. A secondary coil is not even necessary—almost any source of magnetic flux is usable. For example, the watt balance at the National Physical Laboratory uses a permanent magnet as the source of its magnetic flux gradient.

The comparison of the weight of a mass *mg* to the electromagnetic force is called the “force mode” of the experiment. The measurement of the *V/v* ratio is called the “velocity mode.” We can combine these two modes algebraically: Eq. (1) is divided by Eq. (2). This results in the equality

$${\left(mg\;v\right)}_{\text{SI}}/{\left(I\;V\right)}_{\text{EL}}=1,$$ (3)

with subscripts representing the system of units each measurement is made in. The SI subscript represents a quantity measured in SI units, while the EL subscript indicates a quantity measured in terms of the practical electrical units, represented by the Josephson and quantum Hall effects. The combined equation shows a comparison of the SI unit of power—in terms of mass, time, and length, in the numerator—to the electrical unit of power. The equality of Eq. (3) is only valid if the represented electrical unit of power)[*I V*]_E_) is consistent with the SI definition of this electrical quantity.

Because the practical electrical units are linked (through theory) to the values of the fine structure constant, the charge of the electron, and the Plank constant, Eq. (3) can also be considered as a measurement of some product of these constants. For further discussion of these relationships and the role of the watt experiment in supporting the SI, a useful reference is provided in Ref. [9].

## 2. Apparatus

**Fig. 1:**
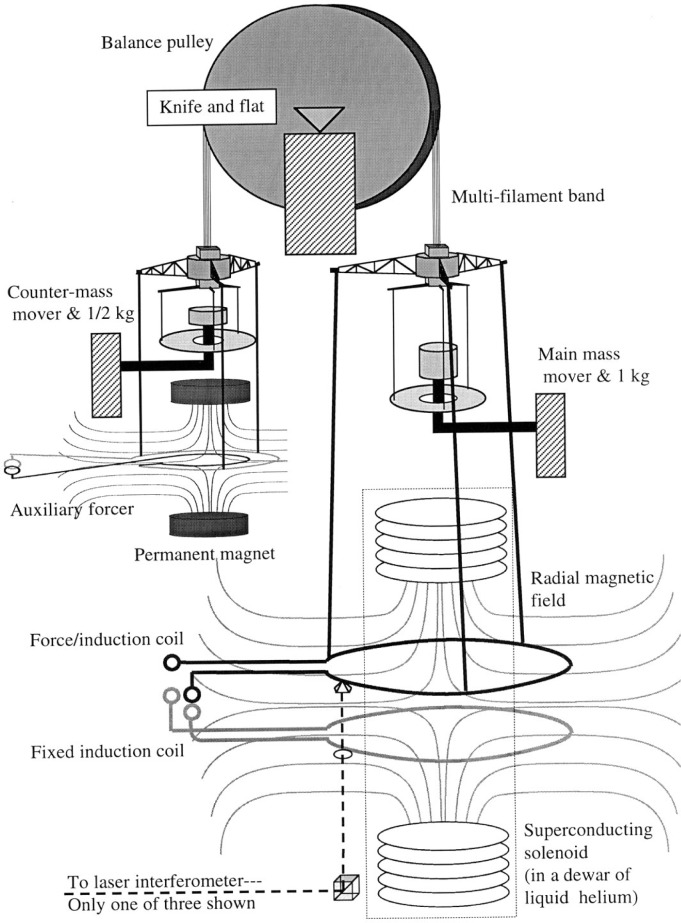
A schematic diagram of the main watt system. Only components relevant to this paper are shown. The drawing is not to scale.

Of interest in this paper are the actual methods that we use to perform the measurements involved in the watt experiment, and their susceptibility to hysteresis errors. A schematic diagram of the watt apparatus relevant here is shown in Fig. 1.

### 2.1 The NIST Watt Balance Apparatus and Measurement Method

The entire balance portion of the experiment is housed in a vacuum chamber at roughly 0.5 Pa. The heart of the balance is a pulley wheel of roughly 30 cm radius, supported by a knife edge pivot at its geometric center. The weights on either side of the pulley hang from multi-filament bands of wire, putting a net load of 610 N on the pivot. The force/induction coil is supported on one side from three rods, and on this same side is a support for a standard 1 kg mass. A counterweight and auxiliary motion control system hang from the opposite side. Electrical connections are made with 40 thin copper wires that are held near the pivot of the pulley.

The source of the magnetic flux gradient is a superconducting solenoid housed in a dewar filled with liquid helium (not in the vacuum system). The solenoid produces a radial magnetic flux density at the force winding. A fixed coil that does not hang from the balance is located in the same field region, and is rigidly connected to the dewar containing the solenoid. The velocity of- and voltage across- the force coil during the velocity mode are measured with respect to the fixed coil, reducing electrical noise.

During the force measurement, current is servoed through the force induction coil to maintain the angular orientation of the pulley in one position. A mass mover system places a tare weight of 0.5 kg mass on the countermass side of the wheel, requiring a current of 10 mA in the force induction coil to keep the wheel from rotating. This current is measured as a voltage drop that is generated as it passes through a resistance calibrated against the quantum Hall resistance. The voltage is measured directly against a Josephson voltage standard.

When a 1 kg mass is placed on the main side of the balance, the current in the coil must reverse. The symmetry of the current reversal around zero current is important. It minimizes the effect of offsets in the voltage integrator, and causes the magnetic force between the force coil and linearly susceptible materials to drop out of the measurement. Finally, it eliminates the change in heat dissipation by the main coil between mass on and off states. Note that the tension in the band does not change between the mass-on and −off states, although the tension in the rods supporting the force coil does.

In the velocity mode the voltage across the force coil is compared to the Josephson volt with a digital volt meter. The measured voltage difference is used as an error signal in a servo loop—the angular speed of the wheel is controlled to produce a constant voltage across the coil. Generating 1 V requires a speed ≈2 mm/s. The velocity of the coil is measured with a laser interferometer. The *V/v* ratio is measured over 8 cm, requiring an angular rotation of ±10° of the wheel. This is a very large range of motion for a balance, which we only accept because our wheel and pivot design solves some other important problems. The wheel system generates a very smooth velocity that can be aligned very well with vertical, reducing undesired coupling of the watt measurements to horizontal magnetic flux gradients.

The auxiliary motion control system is used to control the pulley during the velocity mode. It consists of a coil acting on permanent magnets. Only the coil is attached to the balance. Neither the coil nor the permanent magnet arrangement have a net dipole moment, and both are far from the coil (≈15 auxiliary coil radii away); they only couple to the force coil weakly. The auxiliary coil is opened before the force measurement begins.

After a single voltage/velocity ratio measurement the balance is servoed for a short time (≈1.5 min) with the wheel at ±10°. This time is used to damp out vibrations in the coil, to read temperature sensors, and to save data. Then the direction of the velocity is reversed, as is the Josephson voltage, once again ameliorating the offset effects of contact voltages and thermal voltages. Force and velocity measurements are alternated at roughly a 1/2 hour time interval.

### 2.2 Bands

The multi-filament bands that hang from the main watt balance pulley are made with a non-magnetic alloy of platinum (48 %) and tungsten (52 %). The multi-filament bands roll on and off the wheel much more smoothly than a solid band, providing smoother force coil motion. We use bands with two different diameter wires, 130 μm (0.005) and 77 μm (0.003 in), but we always used the same kind of band for both sides of the pulley. In both kinds of bands approximately 60 wires are used. The wires are separated by 318 μm (0.0125 in). They ride on a polished stainless steel band that was pressed onto the rim of the pulley. Usually bands are heat treated to relieve stress before they are used—the platinum/tungsten alloy resists oxidation.

### 2.3 A Stand-Alone Hysteresis Tester

Because hysteresis measurements are sensitive to many different interactions, we constructed a very simple stand-alone balance to use as a testing platform. Even with a simple system, however, hysteresis measurements are not clean. As discussed by Quinn [10], the dynamics of knife edge pivot/flat interactions are very difficult to model. The dream-like possibility of monitoring the excursions of the balance, and then using available theoretical analysis tools to derive a torque error from them is clearly not practicable.

**Fig. 2:**
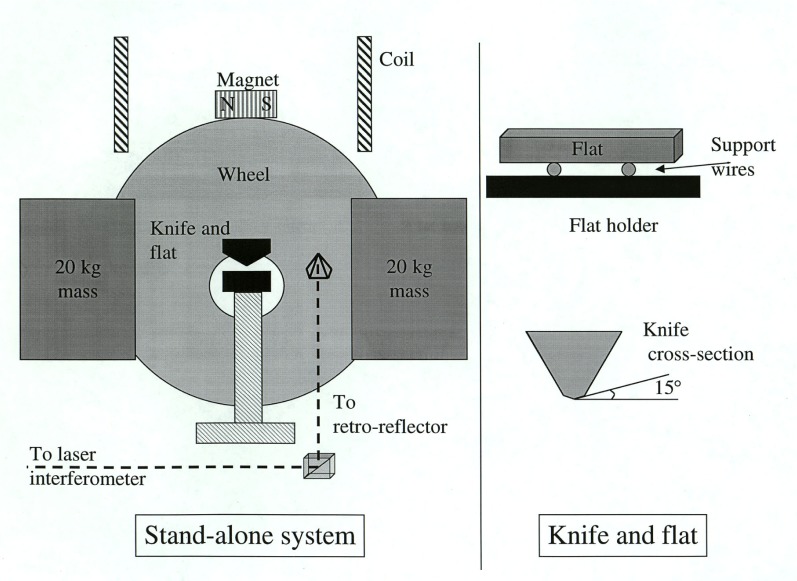
A schematic diagram of the stand-alone system and the knife and flat. The drawing is not to scale.

The stand-alone balance is little more than a rigid load on a pivot, as shown in Fig 2. Two dead weights are firmly attached to the balance, to match the load of the watt apparatus. The orientation of the balance is monitored with a laser interferometer, and controlled with a digital servo that applies a torque through a permanent magnet and coil. The permanent magnet is mounted on the balance. The balance can be servoed over a range of ±175° mrad (±10°), and held to a given angular position to within 500 nrad. This allows measurements of hysteresis torques with a relative standard deviation equivalent to 1×10^−8^ in the watt.

The stand-alone balance is housed in a thermally shielded box covered with aluminum foil to reduce electrostatic forces. During our measurements the thermal gradient in the box was only crudely monitored, and was not controlled. The gradient, in fact, depends on the state of the balance—if a high current is required to servo the wheel, the gradient increases. This makes it very difficult to separate long-term mechanical hysteresis drift from thermal gradient signals. In order to reduce this limitation in the system, we maintained the balance so that only a small current was necessary to servo the balance in our measurement positions (< 0.1 watt dissipation). We accomplished this by making the center of mass of the load correspond to the axis of rotation very well (balance periods of > 120 s). The coils were thermally shielded to further reduce coupling. Our measurements are intentionally short (≈80 s) with respect to the time constant for thermal changes (≈75 min). Because our hysteresis tests are consistently timed, we believe that any hysteresis signal due to thermal gradients would not change from one test to another. For these reasons we believe that the most significant contribution of thermal gradients to our tests would the introduction of small unknown offset in our measurements.

### 2.4 Knife Edges and Flats

The knife material used in the 1998 experiment was a cast alloy of chromium, niobium, cobalt, carbon, and tungsten (TT). This material is nonmagnetic, hard, and tough—not at all prone to brittleness. The knife was used with a boron carbide flat. Both the knife and flat were lapped to optical smoothness. The knife edge had an included angle of 150°, equivalent to a 15° bevel on each face, as shown in Fig. 2. The knife was 7.9 cm (3.1 in) long. The flat was 1.27 cm ×1.27 cm ×10 cm (1/2 in×1/2 in×4 in).

We tested new knives and flats of the same design in a variety of refractive materials: fine- grained submicrometer tungsten carbide (BC6S), hot pressed silicon carbide (SC), and coarse grained tungsten carbide (HF). We coated some of the BC6S and SC pieces with roughly 1 μm thick, diamond-like carbon (DLC), and had some HF parts coated with ≈30 μm to 50 μm thick diamond by a chemical vapor deposition (CVD) process.

DLC and diamond coatings are promising for two reasons. First, diamond has an extremely high modulus of elasticity. We hope that a thick coating may reduce any anelastic strain in the knife or flat. Although it should be possible to have a thicker coating applied, 30 μm to 50 μm was a practical limit for us due to the roughness of the coating. Second, DLC coatings have been shown to have very low coefficients of friction—especially in dry atmospheres [11,12]. If surface interactions are important sources of hysteresis, then the DLC coating would be expected to reduce hysteresis. DLC and diamond coatings could also improve the usable lifetime of the knife edges by reducing damage when an edge is accidentally abused.

For testing, the knives and flats were lapped to an flatness equivalent to that of the components in the 1998 experiment (better than 1 optical fringe on the surfaces adjacent to the edge of a knife and better than 2 fringes over a flat). Although our final stage of polishing used a 1/4 μm grit, which gives an optical quality finish, our edges always had small scratches. We also tested knives lapped with much coarser final grades and saw no clear change in hysteresis. Knives coated with DLC were lapped only before coating. The diamond-coated knifes were lapped before and after coating. The diamond coating proved to be so resistant to lapping that we never achieved an edge or flat that could be tested. We intend to explore the possibilities of having this work contracted to experts.

Before testing, the knife and flat were cleaned in an ultrasonic cleaner (with soap solution), then rinsed with tap water and dried with a jet of dry, clean air. We tested for the effect of contaminating the edge/flat interface with oil and water, and obtained a null dependence result.

Knives were held in aluminum jigs that could be removed from the rest of the wheel. We had two jigs, both of which were interchangeable between the main and the stand-alone system. Although the jigs have slightly different designs, we have not seen any corresponding difference in our hysteresis measurements.

Flats were supported by two copper wires perpendicular to their lengths. The wires were placed to minimize the deflection of the flat when loaded. An aluminum beam supported the wires. Flat deformation is a non-negligible factor in our hysteresis measurements—placing the support wires at the ends of the flat increased hysteresis by a factor of two.

## 3. Hysteresis in the Watt Balance Experiment

A variety of hysteresis mechanisms affect the watt experiment in both its modes of operation. Hysteresis signals affect our primary assumptions about the constancy of the geometry factor *β* between the different modes of the experiment—and thus the validity of the equality of Eq. (3). Only at the level that we can show *β* does not change between measurements will we be able to consider our results reliable.

In the force mode the most important source of hysteresis error arises due to systematic angular motions of the wheel before weighings. When the 1 kg mass is moved onto or off the balance, the pulley tilts because our control servo is imperfect. The direction of the primary excursion of the wheel depends on which way the mass is being moved.

There are several ways that the angular excursion of the wheel might introduce time-dependent or hysteretic torques. A rotation changes the stress on the knife edge, bands, and wheel, possibly resulting in anelastic deformation. Thermal gradients cause deformation of the balance pulley after a rotation, and contact potentials due to friction and material interactions could introduce torques. Further, in the velocity mode the balance is rotated much farther than during the excursions experienced in the force mode. This results in non-linear drifts in the balance zero at the weighing position, which could also skew our results.

Magnetic hysteresis effects can also affect the geometric factor. When current flows through the force induction coil, it perturbs the background magnetic field. If this perturbation changes the magnetic state of nearby susceptible material, then the geometric factor would also change. This could affect either the velocity or force modes of the experiment.

For the new generation of the watt experiment to be a success, we need to reduce and quantify hysteresis effects. In the following sections of the paper we discuss progress towards these ends. Much of the work on mechanical hysteresis was based on earlier work carried out by P.T. Olsen[13].

### 3.1 Mechanical Hysteresis

Our search for lower hysteresis touched upon a variety of solutions—redesign of the wheel balance in part or in whole not the least of them. We have considered using a flexure pivot (which can be modeled much more successfully than a knife edge pivot), magnetically levitating the wheel to reduce the load, or using different pivots for the different modes of the experiment. Nevertheless, we have concentrated on three simple solutions. First, to reduce the systematic excursion size when the mass is moved. Second, to find a materials solution—a knife edge material paired with an appropriate flat that would improve our situation without requiring any redesign of the wheel (“a silver bullet”). Third, to develop techniques to “erase” the memory of the knives, by moving the balance in a systematic motion before weighing. This was attempted without much success in 1998, but still is promising.

#### 3.1.1 Reducing Excursions

We expect a monotonic relationship between excursion size and hysteresis because increasing excursions result in more significant changes in the strain on our components. In the watt experiment, system hysteresis is close to a linear function of excursion size, as can be seen in the data of Sec. 3.1.2. In 1998 the mass mover system had low resolution and was compliant under load, making it difficult to servo. Because of this, the size of the wheel excursions when transferring the mass between the mover and balance were quite large.

We have redesigned the mass mover assembly to reduce excursions. It can now be servoed independently of the wheel with much improved precision, and is much more rigid than the old system. At the time of writing we have not yet had time to perform kilogram weighings. However, we do know that the new mass movers flex by 60 μm (0.2 mrad in the balance angle) when a kilogram mass is placed on them. Thus it is reasonable to expect that we will be able to restrict our balance excursions to less than this level (the mover position can be adjusted at the 2 μm level) during a mass exchange. This is an easy factor of 10 improvement over the 1998 experiment, and we hope for comparable improvements in our uncertainty estimates.

#### 3.1.2 Materials Solutions

In our quest for a materials solution we concentrated on a single type of hysteresis measurement to provide a simple yardstick by which to quantify results. This test consisted of a “three-position” measurement: the standalone balance was servoed to an angular offset from its central position for a fixed time period (80 s), then returned to its central position, where a measurement of the servo torque was made. Then the balance was offset in the other direction and returned. The change in the servo torque at the central position was identified as a single hysteresis value. A series of measurements was conducted at three different excursion sizes: 20 mrad, 40 mrad, and 60 mrad. These excursion sizes were large in order to provide a very clear hysteresis measurement. We also made less extensive measurements at smaller excursions, down to a minimum of 0.7 mrad. We present our results in units of mg cm—a torque normalized to the Earth’s gravity field (the actual torque is in units of mg×*g* cm, where *g* is the local acceleration due to gravity). Roughly 30 mg cm represents a mass error of 1 mg in the force mode of the watt experiment.

Although the precision of our hysteresis measurements is good, the actual hysteresis measurements varied due to the history of a given knife edge and flat. For example, different knives of the same material have different hysteresis, depending on the amount of abuse they have suffered and the original quality of their edges. Typically our hysteresis values span no more than ±10 % for better materials, and quite often are repeatable to better than a few percent. Because of the variable nature of the pivots, we believe that 10 % relative standard uncertainty associated with all of our measurements is a reasonable uncertainty estimate. We have seen that the hysteresis associated with a specific knife/flat combination is stable at the level of ≈1 %, if the system is not disturbed. Thus we will be able to characterize the actual pivot hysteresis in the watt experiment at this improved level.

**Fig. 3:**
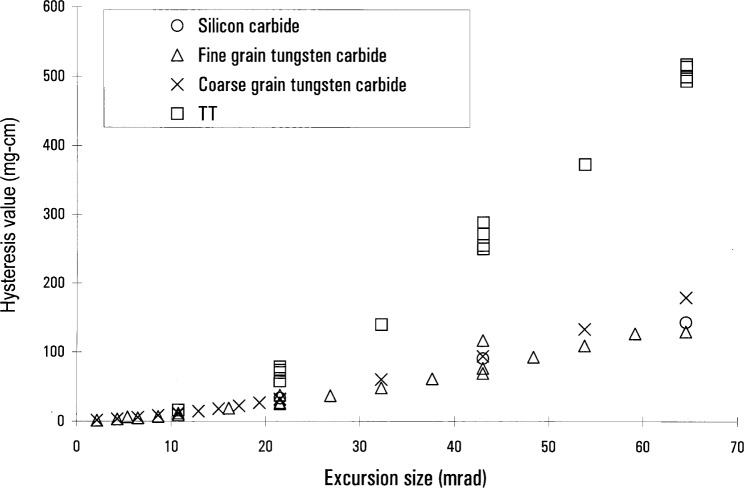
Hysteresis as a function of excursion size for a variety of knife materials on a boron carbide flat.

As shown in Fig. 3, the hysteresis measured depended strongly on the knife material. This is a graph of 3-position measurements made with knives of coarse- and fine-grain tungsten carbide, silicon carbide, and TT. A flat made of boron carbide was used for all measurements. To give an idea of the scale of variations, on this graph we have included data from more than one measurement with TT and fine-grain tungsten carbide knives. Note that the largest hysteresis is associated with TT. Also note the nearly linear relationship between hysteresis and excursion for the carbide knives.

**Fig. 4:**
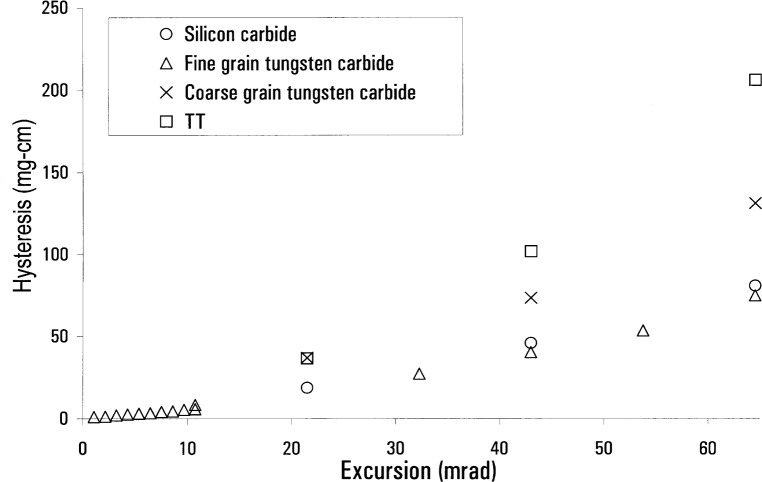
Hysteresis as a function of excursion size for a variety of knife materials on a DLC coated fine-grain tungsten carbide flat.

The dependence of hysteresis on the flat preparation was also very important. Early results showed little difference between using an uncoated boron carbide and an uncoated fine-grain tungsten carbide flat. However there was a big difference when the flat was coated with DLC. Figure 4 is a plot of the hysteresis of various knives on a DLC coated fine-grained tungsten carbide flat. Comparison with Fig. 3 shows a marked reduction. This improvement in hysteresis is very interesting because it indicates frictional and surface effects play an important role in knife/flat hysteresis. If deformation were much more important than surface effects, then we would expect that hysteresis would not vary due to the thin layer of DLC coating. In further support of this idea, the hysteresis seen when using both a coated flat and a coated knife is near the hysteresis with just one coated component (this data not shown). This indicates that the DLC coating does not change the deformation of the components.

**Fig. 5:**
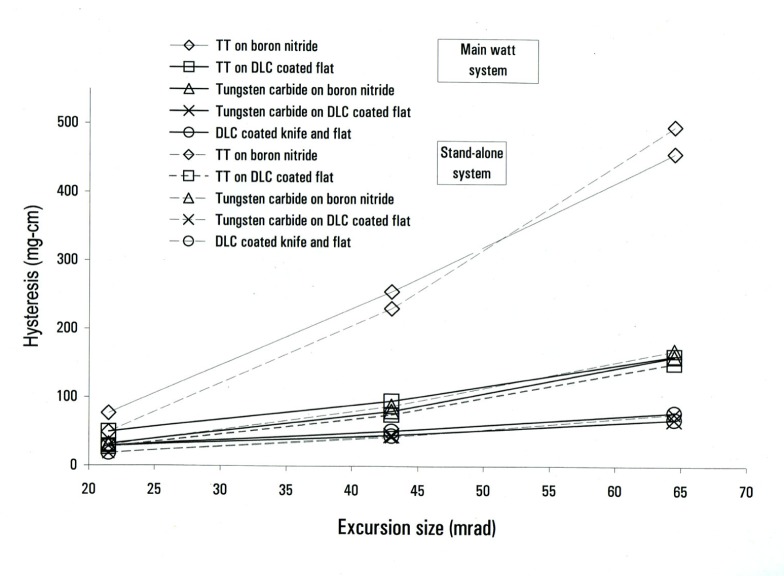
Hysteresis for a variety of knife and flat materials measured on the main watt apparatus (solid lines) and the stand-alone system (dashed lines). Most of the watt apparatus data have been adjusted, as discussed in the text.

We have a reasonable idea of the hysteresis values associated with different knives, flats, and coating combinations. But we have yet to answer the question: do these results, obtained on the stand-alone system, correspond to hysteresis behavior on the main watt system? Figure 5 is a plot of hysteresis data from knife/flat combinations taken on the two platforms. Note that all solid lines represent the watt apparatus measurements, and all broken lines are from the stand-alone balance. The graphic symbols are consistent for each material combination between the two systems. Almost all the data from the watt apparatus has been adjusted to correct for the presence of an additional source of magnetically induced hysteresis that was present when we took the data. When we identified the source, and physically removed it, we characterized its contribution to the overall hysteresis measurement. It was this contribution that was subtracted from our previous results. Only the data for the DLC-coated knife and flat combination were taken without the additional source present.

Because the main system is considerably more complex than the stand-alone balance, the possibility of additional hysteresis sources is not surprising. Although the level of agreement between the two systems shown in Fig. 5 is a convincing statement that the pivot is the largest source of hysteresis in the watt apparatus, we have also tested other possible sources of additional hysteresis. We tested the bands of the wheel, by making measurements with the two types of wire described in Sec. 2.2. We looked for changes in hysteresis correlated to whether the balance was in air or vacuum. No correlation was observed, suggesting that thermal gradients are not affecting our results. This null result also indicates that water layers at the knife/flat interface do not introduce hysteresis. We tested the hysteresis due to the electrical connection by comparing hysteresis before and after the wires were annealed.

#### 3.1.3 Erasing Procedures

We have explored the possibility of “erasing” the memory of hysteretic components of the balance. The idea of erasing is based on the possibility of providing a common history of balance motion before weighing, independently of the direction of the mass transfer. Erasing involves controlling the balance through a series of decreasing excursions, thereby approaching the weighing position in a consistent manner.

To test the effectiveness of erasing in different conditions we used a very simple erasing procedure on the stand-alone system. We used the same three position hysteresis test that was described in Sec. 3, but inserted a 20 s interval for erasing before each measurement at the central position. In this 20 s, the balance was rotated several times between two symmetric positions around the center, and then returned to the center. The balance was always returned to the center from one direction, independently of the direction of the previous excursion. Hysteresis measurements with the erasing procedure were compared to measurements in which the extra 20 s were spent with the balance servoed at its center position.

We found that this erasing technique did reduce hysteresis when the size of the excursion was smaller than the size of the erasing motion. If the excursion was larger than the erasing motion, almost no improvement was seen. We also saw that the erasing technique increased the scatter of our measurements, but not by more than a factor of three. The reduction in hysteresis varied considerably. To within a factor of two, hysteresis was reduced to a value independent of the excursion size. We will need to explore more sophisticated erasing schemes before we will be able to predict an improvement due to these techniques.

The subject of erasing raises the interesting question of the dependence of the hysteresis on the time of the excursion. For long excursions, issues of thermal gradients become important, as discussed in Sec. 2.3. Thus we attempted to study the short time variations of hysteresis. Unfortunately, the torque generated by the stand-alone system servo was so weak that we could only move the balance slowly compared to the actual motions occurring during a mass transfer on the watt system. Thus large excursion measurements with the standalone system were limited to long times when compared to the actual excursions experienced on the watt system during a mass transfer.

The time dependence of hysteresis for excursions lasting between 10 s and 30 s was hidden in the noise of our system. We tested several different knives and flats, including TT, DLC coated carbide, and plain carbide components. Although possible, it is not clear that this null result supports the conjecture of Sec. 3.1.2, on the relative importance of surface to volume hysteresis effects. Even with the null result, we believe that there must be some time dependence. If there were no time dependence, then we could always offset the balance by a large amount in one direction before weighing. This would be sufficient to erase all previous history of the system. In practice, this method was much less effective than the previously described erasing cycle. Clearly there is some time dependence that we will not be able to determine without improving our stand-alone system or exploring with the watt apparatus.

### 3.2 Magnetic Hysteresis and Susceptible Material Signals

We tested for magnetic hysteresis using the main watt apparatus in a series of “zero-field” measurements. These were tests made without current running through the super conducting solenoid. The balance was controlled using the auxiliary force coil, and we searched for signs of hysteresis by recording the auxiliary servo current as a function of the history of the current flow in the force/induction coil.

In “zero-field” when some amount of current *I* is driven through the main induction coil only a small force results ~5 10^−7^ N/mA). This force is due to the interaction of the current with the magnetic flux gradient of the earth *F*_E_ and the induced forces *F*_I_ on paramagnetic, diamagnetic, and ferromagnetic material (attractive, repulsive, and nonlinear, respectively). Upon reversal of the current to −*I*, *F*_E_ will reverse, but *F*_I_ will not. Thus an asymmetry around zero in the force on the balance for ±*I* current flow indicates the presence of susceptible material. Note that without current running through the superconductor, we could drive a great deal of current through the induction coil without creating a force too large to measure. We limited the current flow by our desire to work at reasonably low voltages, and thus only improved our sensitivity to magnetic effects by a factor of three over signals expected during the watt experiment. Also note that without the solenoid field on, we could not study all the effects of susceptible material located on the balance, in particular material in the strong field region.

Merely detecting the presence of susceptible material does not indicate the degree of possible hysteresis errors—sampling the residual magnetic field as a function of the recent history of the current cannot be done with only zero and ±*I* currents: at zero current the coil does not interact with the magnetic field, and the force at ±*I* does not show hysteresis. Offsetting the zero-current state with some current intermediate to ±*I* solves this problem.

**Fig. 6:**
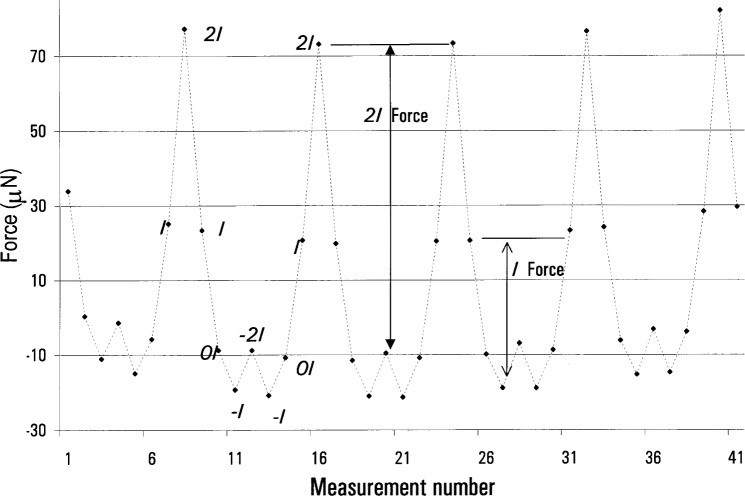
Force generated by the main induction coil with −2*I*, −*I*, 0*I*, *I*, 2*I* current through it. No current is flowing through the superconducting solenoid. These data were taken with soft iron placed near the coil, with *I*≈8mA.

A great deal of information can be obtained by extending the three position measurement to five positions: currents of −2*I*, −*I*, 0, +*I*, and +2*I* are driven through the coil in see-saw order, as shown in Fig. 6. This plots the zero-field servo force to hold the balance in one position for different force induction coil currents. The offset between the average ±*I* and ±2*I* forces and the zero-current force depends on the amount and type of susceptible material present. The difference of the offsets (of the ±*I* and the ±2*I* forces) indicates the nonlinearity of the susceptible material. And the hysteresis of the magnetization can be seen by the difference between the force at either + or −*I*, depending on whether the preceding measurement was zero current or either + or −2*I*, respectively.

Figure 6 shows the raw data from a measurement with iron placed around the coil. The iron was placed above the coil. A positive force represents a force upwards. Thus the 2*I* force is the sum of *F*_E_ and *F*_I_. The force at current *I* includes 1/2 the contribution of *F*_E_, and less than 1/2 of *F*_I_—because the susceptibility of iron is non-linear. The force at zero current represents the balance offset. At a current of −*I*, *F*_I_ does not reverse, thus it cancels, largely, with 1/2 *F*_E_. Finally, with a current of −2*I*, the nonlinear susceptibility results in the induced attractive force of the iron overcoming the downwards −*F*_E_.

**Fig. 7:**
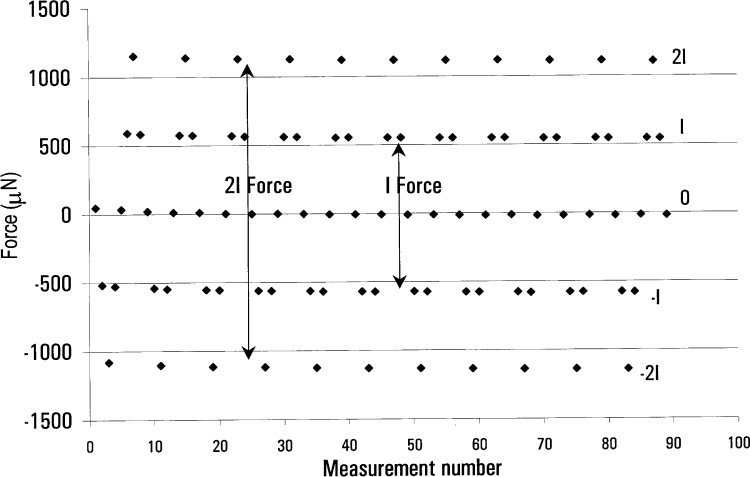
Force generated by the main induction coil with −2*I*, −*I*, 0*I*, *I*, 2*I* current through it. No current is flowing through the superconducting solenoid. These data were taken with the watt apparatus in its standard operating condition, *I*≈8mA

Without additional iron present there is only a slight offset between the zero-current force and the average of the ±*I* and ±2*I* forces, respectively. This indicates the lack of susceptible material near our coil in normal operating conditions. A sample of this data is shown in Fig. 7. Note that the underlying non-linear drift is due to mechanical hysteresis, and is probably the result of temperature gradients. Each data point represents 70 s. The magnitude of the *F*_I_ forces are much larger than in Fig. 6 because of a residual magnetic field associated with persistent currents in the superconducting solenoid.

From the measurements taken of the standard watt system, we can place a worst case limit on the force error due to magnetic hysteresis. The result is satisfying: with the superconductor warm we get a null result with relative standard uncertainty of 2×10^−9^ in the watt. A zero-field voltage/velocity measurement made after the force test produced a geometric factor that agreed with the force measurement within a noise level of 5×10^−9^ relative standard uncertainty—another indication that this is not a significant source of error in our experiment. Force data taken with the superconductor cold gives a null result with standard relative uncertainty of 2×10^−9^ in the watt.

## 4. Further Tests of Magnetic and Hysteretic Error Sources

We have developed several ideas for further tests of systematic effects influencing the watt apparatus. One class of errors we have not yet tested arise from susceptible materials attached to the balance system. These materials can interact with the superconducting field, yet their effects would not show up in our zero-field tests. Thus we will have to work with the field “on”–that is, with current flowing in the superconducting solenoid. We will perturb the magnetic moments of susceptible materials by driving current through the fixed coil. Because the fixed coil is not attached to the rest of the balance system, it will not act directly on the balance. Any forces seen to correlate to the fixed coil current may indicate a 0th order error in the measurement of the geometric factor. Repeating the test with the field off (no current in the solenoid) will indicate any direct attraction between the fixed coil and magnetic material on the balance (not a 0th order error).

We will continue to look for hysteretic magnetization of susceptible materials. Although we put a convincing limit on this source of error, we can approach the problem from a different angle. Because the velocity/voltage measurement is magnetically passive, it will measure the background magnetic field, as well as residual magnetization, without current flowing during the measurement. Thus we will alternate velocity/voltage measurements with the driving of various currents through the main coil. In the zero-field mode velocity/voltage measurements are considerably quieter than with the field up, because stray vibrations of the induction coil do not result in large voltage signals. Thus we expect better signal sensitivity than with the field up.

A different possible source of error was suggested by Fuyuhiko Shiota [14], who works on relating the magnetic flux quantum to the atomic mass unit—an experiment related to the watt balance. He suggested that if current flow in the force induction coil changed the paths of the current in the superconductor wires, either nonlinearly or hysteretically, the equality of Eq. (3) would be voided.

We can test this possibility in a three part measurement. First, we would measure the interaction of the force coil with the fixed coil—much like a separate watt balance experiment in which the fixed coil takes the place of the superconducting solenoid. This would be done with the solenoid at room temperature, without carrying any current. Second, we would cool the superconductor down, and repeat the measurement—still in zero-field. Any difference in the interaction would indicate nonlinear interactions between persistent currents in the solenoid with currents in the fixed coil—and, by extension, with currents in the force coil. Third, current would be driven through the superconducting solenoid. A tare weight would be placed on the mass pan, and current would be servoed through the force coil to maintain the balance angle—as in the force mode of the watt experiment. Driving current through the fixed coil will result in a change in the current flowing in the force coil. This change can be predicted from the results of the first measurement. Any discrepancy between prediction and measurement will identify additional forces linked to changes in the superconducting field. We would vary the current in the fixed coil to identify the superconducting effects as being hysteretic, nonlinear, or merely linear.

## 4. Conclusion

We have studied magnetic and mechanical sources of hysteresis error in the NIST watt experiment. Using a separate balance we have quantified the hysteretic behavior of a variety of pivot materials and preparations. Our conclusions can be summarized as follows:

- A stand-alone system that measures changes in pivot hysteresis has been developed, and can be used to test new pivot designs independently of the watt apparatus.
- Pivot hysteresis is nearly linearly dependent on excursion size.
- Surface interactions are major contributors to pivot hysteresis.
- The pivot is the dominant source of hysteresis in the watt apparatus.
- A DLC coated fine-grained tungsten carbide knife and flat reduce pivot hysteresis in the watt experiment by a factor of five.
- Direct and hysteretic interaction of magnetically susceptible materials around the watt apparatus with currents flowing in the force coil do not introduce significant uncertainty in our results.
- Based on expected reductions in excursion size and pivot hysteresis, we expect a 10- to 50-fold improvement in our uncertainty assignments for hysteresis.

One of our most important conclusions is (iv). The good agreement between the stand alone and watt systems indicates that the pivot is the largest source of hysteresis in the experiment. It follows that further improvements of the pivots will lead directly to reduction in hysteresis uncertainty, and that more research on alternative pivot designs will be of value. Also important is (ii), implying that reductions in excursion size will decrease the overall hysteresis signal in the experiment.

The work presented in this paper leaves a spectrum of questions unanswered. Substantial work remains on improving erasing techniques and quantifying their gains, as well as extending studies on hysteresis dependence on the size and total time of an excursion. Yet the work presented here has improved our understanding of some of the sources of hysteresis in the experiment. We have a clearer idea of the role of excursion size and pivot material preparation on the magnitude of hysteresis errors. We have greater confidence about the importance of magnetic error signals in our uncertainty budget. The NIST watt balance experiment still requires a great deal of research in many different areas before we will be able to present a complete uncertainty budget and a value for the Planck constant with a lower uncertainty. We hope that this paper is a step in the right direction.
